# Safe and secure vehicle routing: a survey on minimization of risk exposure

**DOI:** 10.1111/itor.13130

**Published:** 2022-03-01

**Authors:** Georg E. A. Fröhlich, Margaretha Gansterer, Karl F. Doerner

**Affiliations:** ^1^ Department of Business Decisions and Analytics University of Vienna Oskar‐Morgenstern‐Platz 1 Vienna 1090 Austria; ^2^ Department for Operations Management and Logistics University of Klagenfurt Universitätsstraße 65‐67 Klagenfurt 9020 Austria; ^3^ Data Science @ University of Vienna Universitätsring 1 Wien 1010 Austria

**Keywords:** vehicle routing, safe and secure, survey, consistency

## Abstract

Safe and secure vehicle routing problems refer to the transportation of dangerous (e.g., flammable liquids) or valuable goods (e.g., cash), the surveillance of streets (e.g., police patrols) or other areas (e.g., those within a factory or building), and the response to sudden incidents (e.g., robberies or street disruptions). It thus covers a multitude of models and methods with each having its own objective and constraints, such as unpredictability or risk. We review and classify literature in this field and thereby identify a starting point for researchers in this evolving and practically relevant field. Our study reveals that there are 82 articles that cover aspects related to safe and secure routing, a majority of which were published in the last five years. We classify the articles into five main categories: (i) transportation of hazardous materials, (ii) patrol routing, (iii) cash‐in‐transit, (iv) dissimilar routing problems, and (v) modeling of multi‐graphs. Categories (i)–(iv) elaborate on the problem studied, while (v) provides a general concept based on road network characteristics most commonly found in safe and secure routing problems. Relevant methods and instances, along with their similarities and dissimilarities, have also been discussed in the paper. Furthermore, specific problem characteristics and future research directions are identified.

## 1 Introduction

Vehicle routing is a very well‐studied topic with a lot of ongoing research. However, despite its many classes of problems and importance in real life, safety and security problems (e.g., transportation of cash, police patrols, and transportation of hazardous material) appear to be underrepresented in the literature. An issue possibly faced by the research community is that safety and security problems can take very different forms and might seem unrelated. This results in an even more split community, even though there are knowledge and several overarching concepts between problems to be gained. A reason for this might be the non‐existence of a literature survey, as it could show similarities, synergies, and enable new researchers to have an easier entry into this field of research. We want to address this by giving an exhaustive classification for problems related to safe and secure routing with a special focus on problem characteristics, problem classes, and solution techniques. We only focus on studies in which different variants of vehicle routing problems (VRPs) are modeled and solved using heuristics or exact solution techniques. Related fields, especially the shortest paths problem, do not fit within the scope of our survey and are not considered.

As there is no clear definition of *risk* or *danger* within the studied literature, from hereon we refer to *risk* when a mathematical term is used to measure risk, and the term *danger* when the model is impacted by threats but does not explicitly formulate this (e.g., the danger of being robbed is correlated to the amount of money being transported).

Almost all VRP‐based problems focusing on safe and secure routing are in the field of cash‐in‐transit (CIT), patrol routing, or hazardous material transportation. These three topics are not only very distinct in real life but are also vastly different in terms of risk, objectives, constraints and concerns, and solution approaches. For CIT problems, the danger can be seen as the probability of getting robbed, while specific points must be visited to either pick‐up or drop‐off valuables. Therefore, the routes must be highly unpredictable. Patrolling problems might be similar to CIT problems as they are also focused on visiting specific check points. Once again unpredictable visiting times and routes are essential. However, when patrolling problems focus on covering specific areas over time (e.g., a police patrol being able to arrive within a few minutes after the reporting of an incident) or on preventing incidents by showcasing presence (e.g., military patrols), the danger lies in insufficient coverage of these areas. Hazardous material transportation typically involves visits of specific points. However, in this case, the danger lies in the occurrence of accidents that are generally not caused by malicious outside forces. These problems aim at minimizing expected harms to the population. Predictability of routes or arrivals is not of interest in these settings. Nevertheless, route diversity might be desired to spread the risk among the population.

Additionally, as some common concepts have been identified in several publications on CIT, patrolling, and hazardous material transportation, we want to take a closer look at them even if they are not specifically tailored for the context of safe and secure routing. One of them are dissimilar routing problems, which are some of the strictest models related to dissimilarity. We observe that some extensions have to be made in order to make them fully applicable for safe and secure routing. Similarly, multi‐graph modeling can be found in CIT operations and, as such, could also be applied to some types of patrolling problems. In our study, we give an extensive overview on used data sets in the field. Real‐world instances (RWI) and artificial instances are both commonly used. Depending on the problem they are for arc‐based formulations, simple node‐based formulations, or node‐based formulations using a multi‐graph. Arc‐based formulations allow to consider alternative paths between points of interest (e.g., one being more expensive and another one being cheaper but slower). As these formulations are typically computationally more complex than simple node‐based formulations, a trade‐off between complexity and the advantages of including alternative paths can arise in multi‐graphs.

For our literature study, we searched many library databases that contain major journals on operations research, operations management, management science, etc. This was done using the publicly accessible University of Vienna's platform *u:search* and includes 84 different databases for business and economics (University of Vienna, 2021). The search terms used were pairs of the following two groups' members with the *AND*‐operator (e.g.,“security routing,” “security vehicles,” but not “security safety”):

1. “security,” “safety,” “hazardous material,” “risk,” “patrols,” “guards,” “cash‐in‐transit” and
2. “routing,” “vehicles,” “logistics,” “transportation,” “multigraph,” “dissimilar,” “inconsistent,” “peripatetic”.

However, we decided to only consider journal publications in order to limit our survey to a reasonable number of articles. For each of the initially identified papers, we screened the reference list and selected the articles that were not found in the first step. Finally, we applied a descendancy approach, that is, moving from old information to new by screening all articles that cite one of the papers that we had found to be relevant via *Scopus*. We proceeded until we converged to a final set of publications. This final set consists of 82 articles, the first of which was published in 2007, and more than 50% of them appeared within the last six years (2016–2021).

The remainder of our survey has been organized as follows: Related classifications and definitions are provided in Section 2. Hazardous material routing is surveyed in Section 3. Sections 4 and 5 are dedicated to routing of patrols and CIT, respectively. In Sections 6 and 7, we elaborate on general concepts of dissimilar routes and multi‐graph modeling. A summary is presented in Section 8.

## 2 Classifications and definitions

Security services have a broad field of applications:

- Personal protection
- Transportation of cash, valuables, hazardous materials, or individuals
- Mobile guarding (inspection of different objects in irregular time intervals)
- Plant security services (inspection of objects in irregular time intervals)
- Event security services
- Monitoring (e.g., alarm monitoring and response to elevator calls)
- Custody of private and public buildings or construction sites
- Additional services (e.g., maintenance of security systems, access of control to public buildings, and porter services)

Studies in the field of safe and secure routing can be classified according to the movement of guards and persons or objects, which can be either in motion or fixed. Table 1 provides examples of this classification. Note that some tasks can be assigned to more than one category (e.g., for personal protection, the goal might be to transport persons to a specific place or to take care of a them at a specific place). Only the case with fixed guards and fixed persons or objects does not fall under safe and secure routing due to the absence of routing aspects. Table 2 classifies hazmat, patrol routing, and CIT according to these criteria.

**Table 1:** Classification of security services

		Aspect to be secured	
		Fixed	Moving
Guards	Moving	e.g., plant security services, mobile guarding	e.g., transportation of valuables, hazardous material, or cash
	Fixed	e.g., construction site safety services, custody of a private house	e.g., a guard in a central location monitoring the movement of trucks throughout the city, access control, geo‐locators for children

**Table 2:** Classification of problem classes

	Problem		
	Hazmat (Section 3)	Patrol routing (Section 4)	CIT (Section 5)
Guards	Moving	Moving	Moving
Aspects to be secured	Material (moving) & population (fixed)	Hotspots, facilities, streets (fixed)	Incident during travel (moving) or during service (fixed)

When considering the surveyed literature, some similarities can be observed for hazmat and patrolling problems. In the former, the guards/special vehicles are in motion, with the loaded material being secure and moving as well. However, as risks for hazmat operations are often measured via the population possibly exposed to an incident, parts of the secured objects are immovable. Similar to hazmat, patrol routing involves moving guards. Existing models consider either specific sites or hotspots that are in fixed positions. For literature on CIT operations, a split can be observed. Guards are moving in a vehicle, and valuables are also moving with that vehicle. However, sometimes the assumption is that routes are too unpredictable and, therefore, no attacks will happen, whereas sometimes arrival times are assumed to be unpredictable and no attacks during (un)loading duties at fixed sites are to be expected.

Regarding modeling aspects, the first classification can be made based on the points that must be serviced. These could include street segments that must be patrolled or specific locations such as bank branches. Therefore, they can either be VRPs, arc routing problems (ARPs), or node, edge, arc routing problems (NEARPs). In some settings, VRPs or NEARPs can be simplified by aggregating several locations on the same arc to just one arc, resulting in ARPs or NEARPs. If, for example, the task is to patrol a point by just passing it, aggregating several nodes that are on the same street segment is reasonable. However, this approach might lead to loss in solution quality, if individual tasks are more time consuming, for example, service times, or require specific capacities such as for filling money into automated teller machines (ATMs). In this case, this conversion might be inappropriate as vehicles might be able to service a subset of nodes, but not the complete arc.

An important aspect in safe and secure routing applications are the definition of danger or its inverse, that is, the definition of safety, from which either can be used as a risk measure. In the studied literature, danger or safety are measured as follows:

- path inconsistency (safety, large value desired): nodes, edges, or arcs have to be traversed in inconsistent sequences. This can be achieved through the minimization of the number of traversing times for arcs or edges by varying the routes to be taken between points of service or by varying the sequence in which the services take place (e.g., Duchenne et al., 2012; Talarico et al., 2015b). This measure is commonly applicable in multi‐period models, but typically requires either an arc‐based formulation or a multi‐graph.
- time inconsistency (safety, large value desired): periodical services for the same node, edges, or arc are done at different points in time. This makes it harder to predict the time of service (e.g., Hoogeboom and Dullaert, 2019; Soriano et al., 2020), which is a typical requirement in multi‐period problems.
- population exposure (danger, small value desired): it is commonly found in hazmat publications and combines the likelihood of the occurrence of an incident and its severity for the population (e.g., Pradhananga et al., 2014; Bula et al., 2017), typically via a sumproduct.
- coverage (safety, large value desired): patrol routing problems often aim at covering a large area. Whether a point is covered is usually decided by if it can be reached within a certain time span or if it is within a certain distance (e.g., Capar et al., 2015; Sun et al., 2018).
- detectability (danger, small value desired): surveillance operations such as unmanned aerial vehicles (UAVs) typically do not want to risk being detected under radar and shot down. This can be found in military patrol routing (e.g., Cao et al., 2019; Margolis et al., 2019).
- response time (safety, small value desired): another common goal for patrol routing is to be able to react quickly to any incident. Sometimes average response times are used and, other times, the maximum response time (e.g., Sun et al., 2018; Sun and Wang, 2018).
- expected monetary loss (danger, small value desired): for CIT operations, the severity of an incident can be vastly different depending on whether it took place at the beginning or at the end of a route. When picking up valuables, the possible severity will increase during the trip, while the inverse holds when delivering valuables. Therefore, identical itineraries might have very different risks depending on the nodes serviced and their order. In addition, symmetry will not hold. One possibility is forcing the expected damage of a route to be below a certain threshold (cf. Talarico et al., 2015a) or minimizing it.

It should be noted that path and time inconsistency, which are most common in safe and secure routing literature, are only relevant in multi‐period settings. Hence, models are typically based on a multi‐period formulation where nodes have to be visited more than once within a given time horizon. Note that both *multi‐period* and *periodic* problems cover multiple planning periods. On the other hand, *periodic* implies that the periods are similar, whereas *multi‐period* refers to the general case. Therefore, from here on, we use the term *multi‐period model or problem*.

In addition to the classification presented above, problems can be classified based on the number and characteristics of objectives. We might, for instance, differentiate between (i) minimizing the maximum risk among all routes or (ii) ensuring that risk on each route is below a certain threshold. We identify four types of objectives, namely, (i) single objective, (ii) weighted multi‐objective, (iii) lexicographic multi‐objective, where objectives can be ranked on the basis of priority, and (iv) Pareto‐based multi‐objective, where no priority ranking can be made, but several (non‐dominated) solutions are identified (cf. Fröhlich et al., 2020).^1^

Note that most of these classifications are not limited to safe and secure routing and are suitable for most routing problems.

## 3 Hazardous material transportation

In this section, we survey literature on hazardous material transportation and identify future research directions in this field.

### 3.1 Literature review

**Fig. 1:**
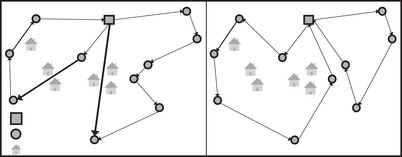
Illustration of a hazardous material VRP. On the left part, only travel distance is considered while routes on the right side are such that travel distance and population risk exposure are also taken into account. The thickness of the arcs indicates the level of population risk exposure.

The majority of studies on hazardous material routing solve the adapted versions of VRPs or capacitated VRPs, and typically try to minimize the danger of population exposure. Generally in these models, edges between two nodes are associated not only with travel cost but also with a predetermined danger level. Solution algorithms are used to generate routes that minimize total travel time and risk exposure. In Fig. 1, we have provided an example of a VRP of hazardous materials, showing the change in the solution if risk in form of population exposure is considered.

Table 3 gives an overview of the studies contributing to the field of hazardous material vehicle routing. We classify them according to (i) the underlying planning problem, (ii) risk measurement method, (iii) the solution method, and (iv) additional characteristics. Furthermore, the instances used are listed. However, as not many are shared, more detailed descriptions are presented in Appendix A.1

**Table 3:** References and basic characteristics for hazardous material routing (see Appendix A.1 for an explanation of the instances)

Reference	Problem	Risk measure	Method	Additional	Instances
Androutsopoulos and Zografos (2010)	2‐SPPTW	Population exposure	Labeling algorithm	Time‐dependent	Art. inst.
Androutsopoulos and Zografos (2012)	2‐CVRPTW	Population exposure	Insertion algorithm	Time‐dependent	Art. inst.
Bula et al. (2017)	HF‐CVRP	Population exposure	VNS	Heterogenous fleet	Art. inst.
Ma et al. (2012)	VRPTW	Via link capacity constraints	TS	Link capacity	sol (modified)
Mohri et al. (2020)	Stackelberg	Population exposure	ALNS		RWI (Singapore)
Pradhananga et al. (2010)	2‐VRPTW	Population exposure	ACO		sol
Pradhananga et al. (2014)	2‐VRPTW	Population exposure	ACO		RWI (Osaka)
Tarantilis and Kiranoudis (2001)	CVRP	Population exposure	Threshold accepting		RWI (Athens)
Taslimi et al. (2020)	PLCVRP	Transportation & storage	Heuristic decomposition	No partial pick‐ups	Art. inst., RWI (Dolj)
Zhao and Zhu (2016)	2‐MDCVRP	Population exposure	Tchebycheff method		RWI (Nanchuan)
Zografos and Samara (1989)	3‐LRP	Via link capacity constraints	Exact solver	Link capacity	Art. inst.
Zografos and Androutsopoulos (2008)	2‐CVRPTW	Population exposure	Insertion algorithm	Compartments	RWI (Thriasian Plain)

2‐, bi‐objective; 3‐, three objectives; ACO, Ant Colony Optimization; ALNS, Adaptive Large Neighborhood Search; Art. inst., Artificial instances; C, Capacitated; HF, Heterogeneous Fleet; LRP, Location Routing Problem; MD, Multi‐Depot; PLCVRP, Periodic Load‐dependent CVRP; RWI, Real World Instances; sol, see Appendix A.1; SPP, Shortest Path Problem; TS, Tabu Search; TW, Time Windows; VNS, Variable Neighborhood Search; VRP, Vehicle Routing Problem.

Tarantilis and Kiranoudis (2001) build routes not in the real space where only travel distances among geographical points are considered, but in a so‐called *risk space*. A risk space is defined by the sum of the products of populations of a geographic object (towns, cities, etc.) and distance‐length between these objects and the point in the graph. The lengths of the projected roads in the risk space are curves computed by an integral. They apply a threshold‐accepting algorithm to generate the time and load‐restricted routes in the risk space graph. Thus, in this work, the risk exposure of the population is explicitly minimized. For testing, they use RWIs based on the city of Athens (Greece).

A bi‐objective model is presented by Zografos and Androutsopoulos (2008). Although the first objective minimizes transportation cost, the second one deals with risk exposure of the surrounding population. The risk for using an arc is the accident probability multiplied by population size. The accident probability is determined using a discrete choice model, where several parameters, such as geometrical features, traffic characteristics, emergency preparedness, and weather conditions are taken into account. The affected population for an arc is estimated via different scenarios, such as jet fire, vapor cloud explosions, and other likely incidents. Therefore, population close to the arc, even when not directly located on it, might be relevant. It is assumed that customers have to be serviced within specific time windows. Hazardous materials are transported in compartments, and each vehicle has several compartments with a given loading capacity. Solutions for RWIs are generated using an insertion algorithm. A very similar setting is investigated by Zhao and Zhu (2016). Again, the authors propose two objective functions, where one minimizes total travel time and the second one minimizes risk exposure. A parameter indicates the population size per edge. Thus, for each edge, the number of persons affected by a hazardous material released along this edge are given. It is assumed that an edge is homogeneous in terms of the size of the affected population along the arc (e.g., not split into high‐ and low‐density areas within one specific the arc). The authors propose a modified lexicographic weighted Tchebycheff method to generate routes for load constrained vehicles and multiple depots with limited space and test it for artificial and RWIs. Androutsopoulos and Zografos (2010, 2012) study an extension of the problem. Like most other authors, they aim at minimizing the total travel time and risk exposure in a bi‐objective manner with time‐dependent travel times and risks as their problem extensions. Androutsopoulos and Zografos (2012), however, also include a fleet of vehicles. In both papers, they aim at creating a set of nondominated solutions. To address the computational complexity, Androutsopoulos and Zografos (2010) use a labeling algorithm that helps generate the k‐shortest nonequivalent paths. With it they manage to generate 20 paths in less than one minute, even for most larger instances. In contrast, Androutsopoulos and Zografos (2012) use a weighted‐sum approach and perform an insertion algorithm that is able to deliver a reasonable number of good solutions within a short computation time.

Pradhananga et al. (2010, 2014) propose an ant colony optimization (ACO) algorithm for a similar two‐objective problem. However, major differences between the two are a limited number of customers serviced per tour in Pradhananga et al. (2010) and the use of real‐world data based on the city of Osaka (Japan) in Pradhananga et al. (2014).

Risk minimization for a routing problem with capacitated heterogeneous vehicles is investigated by Bula et al. (2017). The authors propose a single objective wherein the lengths of arcs influence the level of risk. Thus, the lengths of the routes are not explicitly optimized. For each vehicle, an accident rate is given. The authors assume that risk depends on three factors, (i) the probability of an incident, during which the hazardous material is released, (ii) the impact of such incidents, and (iii) the population exposure inside the predefined impact area of the incident. The probability of an incident is determined based on the truck accident rate, the truck load, the conditional release probability of an accident, and the arc length. Solutions are generated using a variable neighborhood search (VNS) algorithm. Artificial instances are used for testing.

A three‐objective combined location‐routing model is proposed by Zografos and Samara (1989). The objectives are (i) the minimization of risk at disposal sites, (ii) the minimization of routing risk, and (iii) the minimization of travel time. The risk level of disposal sites as well as the risk factors on arcs are assumed to be exogenous. The amount of goods transported on an arc is restricted, and solutions are generated on artificial instances by a commercial solver.

Ma et al. (2012) do not minimize a risk, but they restrict the tonnage transported on an arc. These constraints are motivated by a business project of a Hong Kong hazardous materials transportation company. The authors incorporate these link capacities into a model for the VRP with time windows. A tabu search (TS) algorithm is applied to generate results of artificial instances.

Taslimi et al. (2020) study a novel hazmat problem, where the collection of medical waste is examined. In their case, risk is composed of danger due to transportation, similar to Bula et al. (2017), and danger due to storage. The problem is modeled as a periodic load‐dependent capacitated VRP (CVRP), where a multi‐day schedule for collecting medical waste without any partial pick‐ups has to be created. The authors apply a decomposition‐based heuristic by splitting the problem into single‐period cases and applying column generation. They test this approach on randomly generated instances against a mixed‐integer programming formulation of their problem solved via the commercial solver CPLEX. For instances that CPLEX could solve within 24 hours, they manage to achieve reasonable percentage gaps (between 0% and 6.06%) with their heuristic, with computation times between sub one second and 20 minutes. On larger instances, they deliver better results than CPLEX. The approach is applied to a case study based on the county of Dolj (Romania).

A hazmat problem has also been modeled as a noncooperative Stackelberg game by Mohri et al. (2020). The leader is the dispatcher who aims to minimize risk and travel time, and the follower can be seen as the worst‐case scenario, which aims to maximize the risk with a given amount of road links where accidents may occur. The authors implement an adaptive large neighborhood search (ALNS), and validate it by comparing it for a case study based on the city of Singapore (Singapore), to the nondominated solutions obtained by the commercial solver GAMS. With a time limit of one hour, the heuristic performs only marginally worse on the largest instances that GAMS can still solve. They also examine the trade‐off between the objectives and the impact of several other factors, such as the Stackelberg game characteristics, safe waiting arcs, more diverse routes, and different vehicle fleet sizes.

### 3.2 Future research directions

Studies on hazardous material vehicle routing are very consistent with respect to the modeling of risk. All risk‐based studies listed in Table 3 incorporate risk by including a measure for population exposure into the objective function, and can be considered risk‐neutral; meaning indifference between two distributions with the same expected values. However, most decision makers are risk‐averse (cf. Erkut et al., 2007) and favor high‐probability low‐consequence events to low‐probability high‐consequence events. Therefore, it is desirable to model risk closer to decision makers' preferences. This can be achieved by using perceived risk (cf. Erkut et al., 2007) or expected disutility (cf. Erkut and Ingolfsson, 2000). Alternatively, a promising approach could be to consider risk via additional constraints, rather than in the objective function. These constraints could limit the highest consequence events within the decision maker's preferences. For this purpose, risk‐constrained VRPs, as investigated by Talarico et al. (2015b), Talarico et al. (2017b), and Talarico et al. (2017a) for CIT operations, should be enhanced.

Hazardous material routing is closely related to green VRPs (cf. Eglese and Bektaş, 2014; Lin et al., 2014; Bektaş et al., 2019). Many aspects, such as expected disutility or danger, are relevant in both of these vehicle routing applications. However, these occur specifically on the traversed paths in hazardous material routing, whereas carbon emissions are not just restricted to the routes but affect the whole system. Furthermore, hazardous material routing, contrary to green vehicle routing, is usually not concerned with time‐specific travel times, and therefore, little research has been done in this area. This stems from the fact that travel during congestion is seen as too risky due to higher chances of accidents and their severe consequences, should they occur. Several exact solution approaches have been proposed for green VRPs (e.g., Andelmin and Bartolini, 2017). To the best of our knowledge, hazardous material vehicle routing has not been tackled using an exact solution method or a matheuristic (cf. Archetti and Speranza, 2014), which is a hybrid of heuristics and mathematical programming methods. This constitutes a clear research gap. Moreover, multi‐period versions of hazardous material routing problems are not explicitly studied in the the literature, as the only paper discussing the problem (see Taslimi et al., 2020) proposes a solution approach consisting in the solution of single‐period problems. Besides the reduction of danger and high consequence events, a fair distribution of danger within the population might be of interest. This would most likely result in multi‐period versions concerning finding different paths or increasing the usage of multi‐graphs, in which several predetermined diverse paths are considered.

If hazardous material needs special handling (e.g., cooling and security container with very limited lifespan), stochastic models accounting for possible disruptions — mainly traffic jams and accidents — are recommended. As an incident involving such goods is likely to be of high impact, *unreachability*, as discussed in Nolz et al. (2011), could be a valid measure. This measure expresses the likelihood of a solution becoming impossible, independent of the routing. This could happen if time limitations are applied.

Finally, hazardous material routing problems can be seen in the context of the *Sharing Economy*. Carriers might be willing to cooperate with competitors in order to share transportation dangers. Although there are several studies on sharing capacities in carrier collaborations (see Gansterer and Hartl, 2018; Pan et al., 2019), fair and efficient methods to share transportation risks are yet to be discovered.

## 4 Patrol routing

**Fig. 2:**
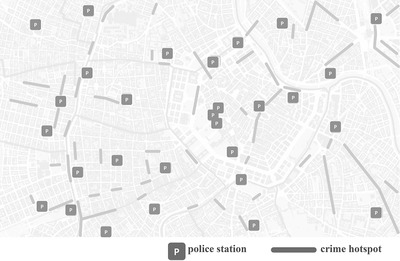
Illustration of a street network with crime hotspots. Patrol routes are expected to traverse as many hotspots as possible; based on Chen et al. (2018).

In this section, we deal with the routing of security personnel, for example, guards and police officers being in charge of surveillance operations or highway patrolling. These problems can be modeled as an ARP if segments of a street network have to be patrolled. An example of a road patrolling problem with hotspots (e.g., streets with high probability of criminal incidents) is depicted in Fig. 2.

**Fig. 3:**
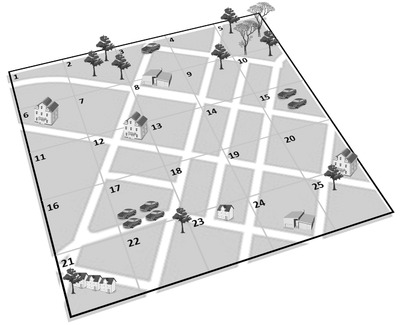
Node‐based patrolling problem where different kinds of objects must be observed; based on Chen (2012).

If objects must be inspected, the patrolling problem is modeled based on nodes. If the objects or street segments are visited once or regularly within a given time horizon, *multi‐period* routing problems have to be tackled. In Fig. 3, we depict a node‐based patrolling problem in which different kinds of objects must be observed.

Patrol routing has several important aspects. Patrols should be visible in order to have a deterrent effect by increasing the perceived danger/chance of detection (e.g., Willemse and Joubert, 2012; Oliveira et al., 2015) or to increase public confidence in policing (e.g., Chen et al., 2018). The routes of the patrols should, however, be unpredictable in order to prevent criminals from being able to side‐step them (e.g., Willemse and Joubert, 2012). Routes are expected to have minimum coverage, which suggests that roads, paths, or regions have to be patrolled evenly and regularly (e.g., Keskin et al., 2012; Willemse and Joubert, 2012; Capar et al., 2015). Patrolling duties should be evenly distributed in order to avoid discontentment and work overload among guards (e.g., Willemse and Joubert, 2012; Oliveira et al., 2015). Finally, routes need to be cost‐efficient as well (e.g., Sun and Wang, 2018).

### 4.1 Literature review

In Table 4, we have listed and classified contributions in the field of patrol routing. We categorized them according to (i) the underlying planning problem, (ii) inconsistency measurement method, (iii) the solution method, and (iv) additional characteristics. Instances used are listed. However, not many are shared, and several real‐world and randomly generated instances are used. Therefore, more detailed descriptions are presented in Appendix A.1.

**Table 4:** References and basic characteristics for patrol routing (see Appendix A.1 for an explanation of the instances)

Reference	Problem	Inconsistency	Method	Additional	Instances
Cao et al. (2019)	M‐MUCR		GA	Risk via radar	Art. inst.
Capar et al. (2015)	TOPTW		Exact solver		RGI, RWI (Alabama)
Chen (2012)	OP	Time	Cross entropy	Dynamic arrivals	RGI
Chen et al. (2018)	ARP	Paths	TS		RWI (Camden), egl
Hajibabai and Saha (2019)	PPRP		CG&GA		RWI (Sioux Falls)
Hsieh et al. (2015)	BGPRP		EA		Art. inst.
Keskin et al. (2012)	TOPTW		LS&TS		RGI, multiple RWI
Li and Keskin (2014)	FSPP		Heuristics	Multiple locations	RWI (Alabama)
Margolis et al. (2019)	MCTPTW		Exact solver & custom additions	Risk via radar	sol (modified)
Oliveira et al. (2015)	MVCTP		Heuristics		TSPLIB (kro, lin, rd), RWI (Vinhedo)
Prischink et al. (2016)	DRPSC	Time	IDR&VND		TSPLIB (burma, st, rd, tsp, gr, berlin, ft, ch)
Saint‐Guillain et al. (2021)	TD‐SS‐VRP‐R		LS		RWI (Brussels)
Sugiura et al. (2015)	VRP		STEN		Art. inst.
Sun et al. (2018)	FSPP		GA	Response time	RWI (Sioux Falls)
Sun and Wang (2018)	FSPP		GA	Response time	RWI (Sioux Falls)
Wallar et al. (2015)	MVCTP		Heuristic decomposition	Risk via radar	Art. inst.
Willemse and Joubert (2012)	ARP	Paths	TS		RWI (Midfield Estate)
Wolfler Calvo and Cordone (2003)	DRPSC	Time	Heuristic decomposition	Alerts	RWI (Milan)
Yassen et al. (2017)	PPRP		Heuristics	Hotspot priorities	sol

ARP, Arc Routing Problem; Art. inst., Artificial instances; BGPRP, Bi‐objective Grid Patrol Routing Problem; CG, Column Generation; DRPSC, Districting and Routing Problem for Security Control; EA, Evolutionary Algorithm; egl, sol, TSPLIB, see Appendix A.1; FSPP, Freeway Service Patrol Problem; GA, Genetic Algorithms; IDR, Iterated Destroy and Repair; LS, Local Search; MCTPTW, Multi‐vehicle covering tour problem with time windows; M‐MUCR, Multi‐base multi‐UAV cooperative reconnaissance problem; MVCTP, Multi‐Vehicle Covering Tour Problem; OP, Orienteering Problem; PPRP, Police Patrol Routing Problem; RGI, Randomly Generated Instances; RWI, Real World Instances; STEN, Space‐Time Extended Network; TD‐SS‐VRP‐R, Time‐Dependent Static and Stochastic VRP with Random requests; TOPTW, Team Orienteering Problem with Time Windows; TS, Tabu Search; VND, Variable Neighborhood Descent; VRP, Vehicle Routing Problem.

Most of the studied problems have either a minimization of costs or a maximization of coverage as their objective. Minimizing a fleet of vehicles could be seen as cost minimization, and reducing total response times is perceived as coverage maximization. Cost minimization is usually the focus when a specific degree of security is desired. However, when a fleet of vehicles or other resources are sunk costs, for instance, due to already fixed wages being paid, coverage can be maximized.

As highway (and freeway) patrolling can be seen as having a few different characteristics than nonhighway (nonfreeway) patrolling, literature shall be split accordingly. Highway and freeway both tend to have longer street segments with less crossings, easily leading to far more expensive and time intensive alternative routes. One of the earliest nonhighway patrolling problems was that of the overnight security service, which was introduced and tackled by Wolfler Calvo and Cordone (2003). The authors present a real‐world case based in Italy where guards not only perform their routine inspections but are also in charge of responding to alert signals, which strongly influence the organization of their duty. The authors propose a solution method based on clustering and routing with the aim to minimize the number of routes. A related study on fleet minimization as its objective is proposed by Prischink et al. (2016). The authors formally introduce the districting and routing problem for security control. Although the former splits all objects into a minimum number of disjoint districts, the latter must be solved for each combination of district and day. Inconsistency is considered for the visiting times. It is assumed that successive visits of an object may overlap, but must be separated by a minimum duration. As for the solution approach, a combination of iterative destroy and recreate operations for the districting and a variable neighborhood descent (VND) are proposed and tested using artificial instances. Willemse and Joubert (2012) investigate the routing of security guards in gated communities. The authors formulate the problem as an ARP, more specifically, as a min–max k‐rural postperson problem or a min–max k‐Chinese postperson problem. The solution, therefore, can be seen as cost minimization. Solutions are generated by a tabu search (TS) that generates sets of routes that are then randomly applied in order to account for unpredictability. RWIs from an estate in South Africa are analyzed. The problem is modeled as multi‐vehicle covering tour problem by Oliveira et al. (2015) with the objective of cost minimization. However, additional balance constraints between individual routes are also included. Several heuristics are used to generate solutions. Experiments based on real‐world data as well as artificial data are presented. Although inconsistency is mentioned as an important aspect, it is not explicitly included in the model. Similar to Willemse and Joubert (2012), it can be assumed that the heuristics generate sets of solutions that are applied for subsequent periods in a random order. A problem expansion of Willemse and Joubert (2012) can be found in Chen et al. (2018). The authors of the latter study formulate the police patrolling problem as min–max multiple‐depot rural postperson problem. Again, a TS is proposed, and real‐world cases where crime hotspots in the city of London (England) must be patrolled are presented. The first coverage maximization found is introduced by Chen (2012). Here, patrol route planning problems are investigated in dynamic environments, in particular, heterogeneous dynamic crime arrivals at individual locations. The model is based on an orienteering problem, and solutions are generated using a cross entropy method on artificial instances. Yassen et al. (2017) have dealt with maximum coverage with hotspot prioritization. Their model is based on a VRP with time windows (VRPTW), and solutions for artificial instances are generated using tailored greedy construction heuristics. A multi‐objective problem, in the form of a bi‐objective grid patrol routing problem, is introduced by Hsieh et al. (2015). Their model includes both the objectives of cost minimization and maximum route coverage. Inconsistency of routes is not explicitly considered. The authors generate solutions using an immune‐based evolutionary approach and test them on newly generated instances. A recent study of a time‐dependent patrolling problem, where vehicles have to be relocated over the day to optimize the responsiveness for any dynamically appearing requests, is handled by Saint‐Guillain et al. (2021). Their objective is to minimize the sum of expected response times for all incidents. They study the problem with and without time‐dependent travel times by comparing a simple wait‐and‐serve method and a local search (LS), where historical data are taken into account on instances based on the city of Brussels (Belgium). Their results indicate that relocating vehicles can yield large improvements.

In the context of highway (and freeway) patrol routing, the early work of Keskin et al. (2012) should be highlighted. The authors work on the state trooper highway patrol problem as a team‐orienteering problem. Solutions are generated by local search and TS‐based heuristics. Using real‐world data from the state of Alabama (USA), the authors give recommendations for (i) critical levels of coverage, (ii) factors influencing the service measures, and (iii) dynamic changes in routes. Improved formulations for this problem and real‐world analysis are proposed in Capar et al. (2015). Due to the improved formulations, larger instances could be solved and a better coverage could be provided. Sun et al. (2018) and Sun and Wang (2018) also maximize coverage by minimizing the overall average incident response time. They study a freeway service patrol problem, which involves patrol routing design and fleet allocation. The authors compare and assess geographically overlapping and nonoverlapping strategies using genetic algorithms (GA) for real‐life instances based on the city of Sioux Falls (USA). Hajibabai and Saha (2019) tackle the patrol routing problem with the goal to minimize the total expected incident response times. A column generation‐based solution technique is used to solve the problem for different station designs. To assess the impact of dispatching scenarios on routing costs, the authors integrate GAs with continuous approximation. They also test this on instances of the city of Sioux Falls (USA) used by Sun et al. (2018) and Sun and Wang (2018). However, Sugiura et al. (2015) deviates slightly, as road patrols — at least in Japan — are in charge of not only preventing accidents by driver control but also of removing objects that have fallen on the routes and repairing potholes. They propose to use smart forecasting methods and statistical analyses for road patrolling services, since, for example, pothole occurrences seem to be relatively easy to predict. Therefore, the authors formulate the road patrolling problem as a VRP and solve it for artificial instances using a space‐time extended network with the aim to minimize the travel times. A bi‐criteria location‐routing problem for patrol coverage of highways is studied by Li and Keskin (2014). They aim to minimize costs, including salaries and costs of routing and facilities, and maximize coverage. In addition, it allows state troopers on patrol routes to have multiple starting and stopping spots and considers that hotspots and temporary station change dynamically. They solve it using real‐world data from the state of Alabama (USA) via a heuristic and compare the results to the commercial mixed‐integer programming solver CPLEX.

Another special case of patrol routing can be found in military applications. Wallar et al. (2015) study a multi‐vehicle covering tour problems, where a fleet of UAVs has to scan a specific area. Risk occurs by UAVs being within the range of detection by sensors, which can be mitigated by flying at a greater altitude, which, however, will lower the data quality of the feed. As such, risk and data quality can be seen as a direct trade‐off. As a solution approach, first, a two‐dimensional routing can be created, ignoring the altitude. After the route is fixed, they adjust the altitude to generate desired trade‐off solutions. Cao et al. (2019) also consider a UAV‐routing problem, a multi‐base multi‐UAV cooperative reconnaissance problem to be more precise. Several UAVs, which may be located in different bases, have to scout a set of targets, and to accomplish this, sometimes multiple UAVs must be used within a very specific time frame. Similar to Wallar et al. (2015), they establish the time within detection range of radars as a risk. Although they aim to minimize risk, they engage in a conversion into costs. This can be done since their UAVs fly at constant speeds. As a solution approach, they propose a GA. Margolis et al. (2019) consider a multi‐objective multi‐vehicle‐covering tour problem within a specific time window by taking risk minimization and coverage maximization as objectives. Here, risk is defined as in the cases above. They determine efficient sets via the ε‐constraint method with an underlying exact solver that is strengthened with labeling algorithms and the use of Lagrangian dual variables.

### 4.2 Future research directions

We observe that route or time inconsistency has been hardly considered in relation to patrol routing, with Chen (2012) being the only exception. This is surprising as unpredictability is regularly listed among the most prevalent issues in surveillance operations. Most studies seem to propose generating several single‐period problems and applying them in a random order for subsequent periods (e.g., Willemse and Joubert, 2012), while others modify time windows to achieve diversity in solutions (Wolfler Calvo and Cordone, 2003). At this point, researchers tackling patrol routing problems could benefit from contributions in CIT, where both time and path inconsistencies are intensively investigated (see Section 5).

In the field of patrol routing, a majority of studies is based on static settings with all necessary information being available. However, it can be assumed for highway and freeway patrolling that incidents as well as sudden changes in traffic conditions (e.g., traffic jams and accidents) occur dynamically, that is, inconsistently. Non‐highway‐non‐freeway patrolling might face similar events, but to a lesser degree and/or with more alternative routing possibilities. Although a few studies include the dynamic aspect of incidents either via dynamic arrivals, alerts, or maximum response times (Wolfler Calvo and Cordone, 2003; Chen, 2012; Sun et al., 2018; Sun and Wang, 2018), they do not consider changes in traffic. Adapting optimization problems by including real‐time traffic data and creating online algorithms, therefore, seem to be a promising extension that could be used in real‐world applications (Borrodin and El‐Yaniv, 2005). Online algorithms are characterized by not having all information available and creating a plan during execution with new incoming information (e.g., a driver executes a part of the route, while the next part of the route is determined). Offline algorithms, in contrast, create a plan ahead of time with simple guidelines how to handle interruptions/alarms (e.g., let the closest vehicle handle the alarm, insert it within the current solution at the best position) and do not perform major recalculations (e.g., use an LP‐model with the new data and execute a metaheuristic). These characteristics allow online algorithms to be more responsive but the limited computation time granted for updates might result in poor solution quality. Therefore, a valid alternatives might be predetermined alternative routes (e.g., a secondary route, if an unexpected traffic jam occurs), which could be used in a stochastic model that is solved offline.

Solution schemes for handling incidents (e.g., reacting to an alarm) and investigation whether these are robust seems to be a promising research question. Robust schemes are able to handle the majority of incidents with marginal changes to costs and security. Simple methods of handling incidents like rerouting just a single vehicle might cause previously well‐covered areas to be less secure. Also, such an approach would be exploitable by faking an incident and then striking somewhere else. By rerouting multiple vehicles, the loss of security could be addressed, but at the costs of an increased complexity that demands more efficient methods.

## 5 Cash‐in‐transit

In this section, we focus on CIT operations, where money has to be either picked up from or delivered to banks, ATMs, etc.

### 5.1 Literature review

The literature review on CIT is split into three parts: (1) time inconsistency, (2) path inconsistency, and (3) other measures. In Table 5, we list and classify relevant contributions to this field. Categorization is according to (i) the underlying problem, (ii) the way inconsistency is measured, (iii) the solution method, and (iv) additional characteristics. Subcategories are made according to the type of inconsistency or other measures used. Although instances are also listed, explanation for the more common ones are found in Appendix A.1.

**Table 5:** References and basic characteristics for CIT‐related work (see Appendix A.1 for an explanation of the instances)

Reference	Problem	Inconsistency	Method	Additional	Instances
Bozkaya et al. (2013)	CVRP‐PD‐TW	path	ARBIPSA		RWI (Izmet)
Duchenne et al. (2005)	m‐PSP	path	B&C		RGI, TSPLIB (burma, gr, fri, bays)
Duchenne et al. (2007)	m‐PSP	path	B&C		RGI, TSPLIB
Duchenne et al. (2012)	m‐CPSP	path	B&C, B&P		RGI, TSPLIB
Ngueveu et al. (2010b)	m‐PVRP	path	ILS, TS	Lower bounds	TSPLIB (bays, fri, gr), A, B, P, vrpnc
Ngueveu et al. (2010a)	m‐PVRP	path	Hybrid‐TS		A, B, P, vrpnc
Ngueveu et al. (2013)	m‐PVRP	path	CG		A, B, P
Talarico et al. (2015b)	kd‐VRP	path			R, V
Zajac (2018)	kd‐VRP	path			A, B, F, X, tai, vrpnc
Constantino et al. (2017)	DisARP	time	MILP, MH		Art. inst.
Hoogeboom and Dullaert (2019)	PVRP‐TW	time	IG‐TS	TSC	sol for CIT, RWI (Netherlands)
Michallet et al. (2014)	PVRP‐TW	time	MS‐ILS	TSC	sol for CIT, RWI (France)
Soriano et al. (2020)	PVRP‐TW	time	ALNS	TSC, MG	sol for CIT, RWI (Vienna)
Yan et al. (2012)	MCNFP	time	MH		RWI (Chungli)
Yan et al. (2014)	MCNFP‐STT	time	MH		RWI (Chungli)
Allahyari et al. (2021)	S‐PDPTW	other	ALNS		sol, Art. inst., RWI (Tehran)
Talarico et al. (2015a)	RCTVRP	other	GRASP, ILS		A, B, F, tai, kelly, vrpnc
Talarico et al. (2017a)	MO‐RCTVRP	other	ILS & Promethee II		L, R
Talarico et al. (2017b)	RCTVRP	other	ACO‐LNS		R, V, O, S
Tikani et al. (2021)	TD‐RCTVRP	other	DP, heuristic DP, GA	Stochastic, MG	RWI (Isfahan)
Radojičić et al. (2018)	RCTVRP	other	F‐GRASP & PR		R, V, O, S

A, B, F, L, O, P, S, R, V, X, kelly, sol, tai, vrpnc, TSPLIB: see Appendix A.1; ACO‐LNS, Ant Colony Optimization with Large Neighborhood Search; ALNS, Adaptive Large Neighborhood Search; ARBIPSA, Adaptive Randomized BI‐objective Path Selection Algorithm; Art. inst., Artificial instances; B&C, Branch and Cut; B&P, Branch and Price; CG, Column Generation; CVRP‐PD‐TW, Capacitated VRP with Pickup and Delivery and Time Windows; DisARP, Dissimilar Arc Routing Problem; DP, Dynamic Programming; F‐GRASP & PR, Fuzzy GRASP & Path Relinking; GA, Genetic Algorithm; GRASP, Greed Randomized Adaptive Search Procedure; IG‐TS, Iterated‐Granular TS; IG‐TS, Iterated Granular Tabu Search; ILS, Iterated Local Search; kd‐VRP, k‐dissimilar VRP; m‐CPSP, m‐Peripatetic Capacitated Salesperson Problem; m‐PSP, m‐Peripatetic Salesperson Problem; m‐PVRP, m‐Peripatetic Vehicle Routing Problem; MCNFP, Multi‐Commodity Network Flow Problem; MG, Multi‐Graph; MH, Matheuristic; MILP, Mixed Integer Linear Programming; MO‐RCTVRP, Multi‐Objective RCTVRP; MS‐ILS, Multi‐Start ILS; PVRP‐TW, Periodic Vehicle Routing Problem with Time Windows; RCTVRP, Risk‐constrained Cash‐in‐Transit Vehicle Routing Problem; RGI, Randomly Generated Instances; RWI, Real World Instances; S‐PDPTW, Secure Pickup and Delivery Problem with Time Windows; TD, Time‐Dependent; TS, Tabu Search; TSC, Time Spread Constraints.

#### 5.1.1 Time inconsistency

**Fig. 4:**
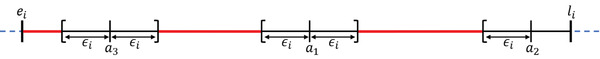
Time inconsistency expressed as time spread. $a_{1}$, $a_{2}$, $a_{3}$ are service times in previous periods; the red lines depict the times that would meet the time inconsistency; based on Hoogeboom and Dullaert (2019).

One possible concern of CIT operations is the diversification of service times for periodically visiting customers. Current studies model this as a constraint by restricting customer service times within a certain interval (e.g., Michallet et al., 2014; Hoogeboom and Dullaert, 2019; Soriano et al., 2020). Figure 4 depicts an example for time inconsistent routing. The time window for a customer i is given by $e_{i}$ and $l_{i}$ for the earliest and the last start of service, respectively. The service times of previous periods are denoted by $a_{1}$ to $a_{3}$ with the surrounding $\varepsilon_{i}$ time units currently not being sufficiently diverse.

**Fig. 5:**
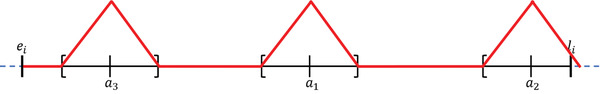
Penalty for violating time inconsistency as expressed by time spread. $a_{1}$, $a_{2}$, $a_{3}$ are service times in previous periods; the red line depicts the amount of penalty when servicing at a specific point of time; based on Michallet et al. (2014).

Michallet et al. (2014) model such a CIT problem as a periodic VRP with time spread constraints on services (PVRP‐TW), in which a set of customers must be serviced in multiple periods at a minimal cost. They assume a limited fleet with limited vehicle capacity. Waiting time is not allowed, and it is assumed that information on required customer services is available *a priori*. A mathematical model is provided and solved by multi‐start iterated local search (ILS). Due to the large amount of constraints, the authors allow for infeasible solutions, which are penalized. The structure of the penalty for the time spread constraints is important. The penalty increases with the closeness to the forbidden point in time (see Fig. 5). A modification of the problem is presented by Hoogeboom and Dullaert (2019). The authors assume that only the customers of the current day are known. This changes the problem to a sequential solution of VRPs with multiple time windows. They also allow their heuristic — an iterated granular TS — to create solutions violating the constraints. However, when comparing to Michallet et al. (2014), minor changes for the penalty evaluation due to time spreads are imposed. Although both study a version in which waiting is not allowed and the departure time is set in a manner that the penalty is minimized, Hoogeboom and Dullaert (2019) also consider a departure as early as possible and a version in which waiting is allowed. In addition, Hoogeboom and Dullaert (2019) provide a computationally more efficient implementation. A related work is done by Soriano et al. (2020). The authors use a problem formulation similar to Michallet et al. (2014) and solve it by applying an ALNS with further intensification of promising solutions through a VND. Additionally, they examine the effect of introducing multiple alternative paths to the problem. As waiting itself is not allowed, it might help to artificially wait by simply taking a longer route, which is similar to a penalty in other models. Their results show that a multi‐graph approach can be beneficial as they have improved previous results.

In Yan et al. (2012), a PVRP‐TW is studied for instances based on Taiwan's district of Chungli. The authors, however, assume that demands are known only one day in advance and allow for the penalization of waiting times. The problem is modeled as a multiple commodity network flow problem that includes risk with two sets of parameters $\beta_{1},\text{…},\beta_{n}$ and $\delta_{1},\text{…},\delta_{n}$. Parameter $\beta_{i}$ indicates the required difference in arrival time at the nodes compared to the arrival times i periods earlier, while parameter $\delta_{i}$ is the maximum similarity between the current routes allowed as compared to the routes i periods earlier. The difference to recent solutions is considered more important (i.e., they have a higher weight) than earlier solutions. The authors solve the integer linear programming formulation (ILP) by splitting it into sub‐periods. These are sequentially solved from the earliest to the latest, while decisions are fixed regarding earlier service arcs. This model is extended in Yan et al. (2014) by taking stochastic travel times into consideration as well as introducing penalty costs for unanticipated late arrivals. The nonstochastic and stochastic models are tested against each other for the stochastic case by generating multiple scenarios. The authors show that the stochastic model yields superior solutions.

A dissimilar ARP (DisARP) is studied in Constantino et al. (2017) where dissimilarity is also defined on a time‐basis — but later handled differently. The DisARP consists of a mixed capacitated ARP, which is extended to multiple days. Each day is split into multiple periods (or time windows), while identical periods of neighboring days may not share the service of any arc. Each period is required to have a minimum number of services. Furthermore, periods as well as services do not have actual times attributed to them. Since the time inconsistency is remodeled as route inconsistency, it is not clear whether it is to be considered time‐based or not. To solve this problem, Constantino et al. (2017) use a matheuristic, which identifies tours via a linear model and later composes a solution out of multiple tours. First, they create a set of initial solutions for single days, from which they then choose a set of solutions for the set of days. Thus, the total routing cost is minimized, while similarity constraints are respected. The exact model is compared against the matheuristics on newly generated instances. For larger instances where the exact model reaches a time limit, the matheuristic delivers better results.

#### 5.1.2 Path inconsistency

Several very different approaches for path inconsistency in CIT can be found in the literature. The strictest of them might be m‐peripatetic models, such as the m‐peripatetic traveling salesperson problem (m‐PTSP) in Duchenne et al. (2005, 2007, 2012), and the m‐peripatetic VRP (m‐PVRP) in Ngueveu et al. (2010a, 2010b, 2013). These consist of a base problem such as the traveling salesperson problem, which has to be solved for a number of m periods without reusing edges or arcs. All these studies use artificial instances.

In Duchenne et al. (2005), undirected m‐PTSPs are mathematically modeled in 2‐index and 3‐index formulations. Since the 2‐index formulation is a relaxation of the 3‐index formulation, repair methods help guarantee that feasible solutions are used. Duchenne et al. (2007) expand on this by introducing more inequalities for stronger cuts. Furthermore, the decomposition technique for the 2‐index formulation is improved. Both papers show that their 2‐index approach delivers better results. The undirected m‐PTSP is relaxed to an m‐capacitated peripatetic TSP in Duchenne et al. (2012). It allows edges and arcs to be traversed multiple times during the *m* periods as this might be more applicable to many real‐world security problems. The authors use three different mathematical models in conjunction with appropriate solution methods: a 3‐index formulation with a branch‐and‐cut (B&C) algorithm, an edge‐edge formulation with a B&C algorithm, and a set‐partitioning formulation with a branch‐and‐price (B&P) algorithm. The latter two models address the problem of symmetry for m‐peripatetic problems, meaning that two solutions in the search space that are similar are exchanged. They achieve the best results with their B&P using the set‐partitioning formulation.

Ngueveu et al. (2010b) can be seen as the starting point for the follow‐up work presented in Ngueveu et al. (2010a, 2013). Two lower bounds for the m‐PVRP are given by solving the relaxations of the m‐PVRP. Additionally, upper bounds are heuristically determined via an ILS and a guided/granular TS with diversification. In Ngueveu et al. (2010a), the guided/granular TS with diversification is hybridized with linear programming in the form of a b‐matching problem, which is a relaxation of the m‐PVRP. This approach delivers good results not only for the m‐PVRP but also for two special cases, namely VRP and m‐PTSP. In Ngueveu et al. (2013), better lower bounds are created using a column generation‐based bounding procedure, in which multiple heuristics are used for generating more efficient cuts. Furthermore, they develop a B&C on edge‐based formulations as an exact solution approach.

A less restrictive definition for path inconsistency and security can be found in Bozkaya et al. (2013). The authors study a capacitated VRP with pickups, deliveries, and time windows. The aim is to minimize a weighted objective that consists of cost and risk. Risk is classified into two different types, namely socio‐economic risk and usage‐based risk. Both types are defined on the paths taken. Socio‐economic risk is seen as a fixed risk, depending on the traversed neighborhood. Usage‐based risk, on the other hand, is determined by how often a certain arc is used. The authors update usage‐based risk similar to an exponential smoothing approach with a weighted sum of the previous usage‐based risk and the number of times that an arc was used on the previous day. For solving their problem, they first generate a set of dissimilar and shortest paths between all points that need to be serviced. The days are solved sequentially by first selecting one of the alternative paths between each pair of points for each direction and then applying an ALNS. Alternative paths are selected randomly with probabilities being inverse to the current risk. After having a solution, a time adjustment algorithm is applied in order to diversify arrival times at demand points. During this step, the sequence of the routes cannot change, resulting in the total distance and the risk of the route not changing, thereby ensuring that the total objective value remain the same. However, it should be noted that, even though the risk is path‐based, time‐based risk is considered within post‐processing. Experiments are conducted on RWIs.

**Fig. 6:**
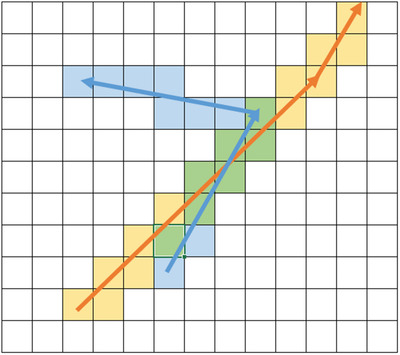
Spatial similarity of two routes according to grid units. Yellow and blue grid units are part of just the orange and blue path, respectively. Green grid units are part of both.

The k‐dissimilar VRP (kd‐VRP), which is based on path dissimilarity, can also be found in the security context. It aims at finding a set of k solutions and solving a VRP that minimizes the costs of the most expensive route, while all solutions must be sufficiently dissimilar. Talarico et al. (2015a) compare all pairs of routes from two solutions and set the similarity to the highest level between the pair of routes. Similarity of routes is determined by the proportion of the costs of shared edges. This measure, as well as the use of artificial instances, can be found in Zajac (2018) who additionally use a spatial measure by taking the proportion of shared grid units for the similarity between routes. This requires the imposition of a grid onto the map of the investigated area. A grid unit is part of a path if the path somehow touches any border of the grid unit. Figure 6 depicts such a grid with two paths.

#### 5.1.3 Other aspects

**Fig. 7:**
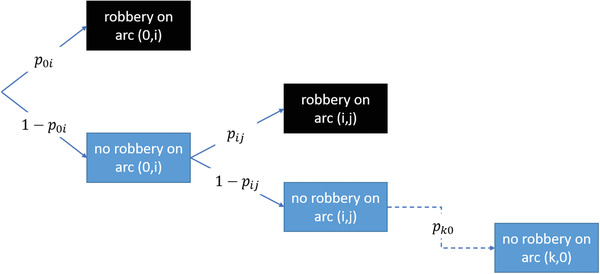
Probability tree for being robbed. $p_{xy}$ indicates the probability of being robbed while traversing arc xy; based on Talarico et al. (2015a).

**Fig. 8:**
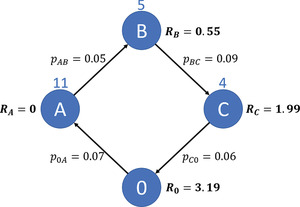
Risk in RCTVRP. $R_{i}$ depicts the cumulative risk until arriving at node i in the case of a CIT pickup problem. $p_{ab}$ depicts the probability of being robbed from node a to b.

An alternative approach for inconsistency based on paths can be found in the risk‐constrained CIT VRPs (RCTVRP), for example, Talarico et al., 2015a, 2017a, 2017b; Radojičić et al., 2018. The RCTVRP is a remodeled CVRP where routes are limited not by a vehicle's capacity but a maximum risk that is allowed per route. Conceptually, the risk of a route where the consequences of being robbed and the likelihood of being robbed are taken into account. Since a route would end after a robbery, the probability tree can be depicted as shown in Fig. 7. However, since the probability of being robbed multiple times during a route can be neglected, a nonconditional probability is used. This reduces the probability for an arc b with a set of prior arcs A from $p_{b}\prod_{a\in A}\left(1-p_{a}\right)$ to $p_{b}$. Figure 8 illustrates the risk for a given route from the depot 0 to nodes A, B, and C, and back. The numbers above the nodes and arcs correspond to the money being picked up and the probabilities of a robbery, respectively. The terms $R_{i}$ indicate the risk at node i.

In Talarico et al. (2015a, 2017b), heuristics for the RCTVRP are developed. In Talarico et al. (2015a), combinations of four construction heuristics with two local search frameworks, one being a greedy randomized adaptive search procedure and the other being an iterative local search, are examined. In Talarico et al. (2017b), the authors develop a combination of ACO and large neighborhood search (LNS). An ACO generates the initial solution via a TSP, where the risk constraint is relaxed, which is then split into a solution respecting the risk constraint. The LNS tries to improve the solution. Additional diversification is generated via perturbation, starting from a new initial solution. Thus, the results presented in Talarico et al. (2015a) are improved. Further improvements are presented in Radojičić et al. (2018). The authors use a fuzzy GRASP in combination with path relinking and present their approach with and without *fuzzification*. Their fuzzy algorithm yields better results regarding average solutions, average iterations, average computation time, and a number of best‐found solutions. A combination of time‐dependent travel times and their uncertainty is studied by Tikani et al. (2021), with risk being defined similarly to Talarico et al. (2015a). They assume that arcs might get congested, increasing travel time and, consequently, danger. Different arcs might have different probabilities of congestion, which does not always result in the same path from one node to another being the optimal choice. Therefore, a multi‐graph is applied. They test an exact two‐phase dynamic programming‐based algorithm, a heuristic dynamic programming method, and a hybrid method based on GAs and dynamic programming. Using a case study based on the city of Isfahan (Iran), they compare their methods on a deterministic and a stochastic case. They show that their heuristic yields promising results as it manages to solve many of the instances that could be solved by the exact version to optimality. An improvement by hybridizing the GA is also shown. Based on their case study, they also deliver the value of perfect information, which they deem as important but difficult to obtain.

Talarico et al. (2017a) study a multi‐objective RCTVRP using RWIs. For the minimization of the maximum risk, the second objective, a local search similar to Talarico et al. (2015a) is applied. The authors propose a weighted sum of the objectives. The weights are set during the search according to the decision makers' preferences and the current set of solutions. A weighted multi‐objective secure pickup and delivery problem with time windows is studied by Allahyari et al. (2021). They try to minimize the sum of total costs and risk stemming from the routes with risk being composed from arcs that have been used multiple times. A similar methodology is true for arrival times and total travel time. Similarly to Talarico et al. (2015a), the valuables within the vehicles are also taken into account. After providing a mathematical model and noting its complexity and impracticability for larger instances, they approach it via an ALNS. They show that their ALNS outperforms their CPLEX‐formulation under a time limit of three hours and that a further intensification with improvement neighborhoods and a departure time optimization yield great benefits. Furthermore, they validate their method by comparing with other heuristics for the Solomon instances (Solomon, 1987) and also supply a case study based on the city of Tehran (Iran).

### 5.2 Future research directions

A lot of different models and assumptions have already been proposed for CIT problems. However, it seems that in most cases, either a type of time inconsistency or path inconsistency is examined with both advantages and disadvantages. Investigating whether there are strong positive correlations between some diversity criteria (e.g., optimizing criterion 1 results in a good value for criterion 2), which might not be symmetrical, might be of great interest. It could allow future models to focus on a smaller number of diversity criteria and, thus, could result in overall better comparability of methods.

Current models focus mostly on a single criterion. Decision makers, however, often consider multiple criteria or would prefer criteria, besides their primary one, to also be optimized. Lexicographic approaches, as in Bozkaya et al. (2013), could sometimes be included at a very low computational cost. Bozkaya et al. (2013) aim at minimizing costs and maximizing route inconsistency. They fix the routes and only diversify the arrival times. This computationally simple adaptation improves the time inconsistency, while not altering route inconsistency or costs. As an alternative to lexicographic approaches, methods used for CIT‐problems could be extended to generate Pareto sets (sets of nondominated solutions, where no solution exists that is better regarding at least one objective and equally good regarding all others). This would not only be of interest for models with multiple types of inconsistency but also for models considering just one type, as it is also not trivial to combine costs and security as a weighted sum.

CIT operations also face the problem that it is difficult to measure whether any incidents have actually been prevented due to unpredictable routing. This is further exacerbated by the fact that robberies are rare types of events, which makes it challenging to use measured real‐life data for creating and validating models (cf. Prieto and Bishop, 2016). Therefore, it might be interesting to develop models, where the objective is to perform robberies and that can dynamically adapt to the CIT routes. These findings can then be used in the original models. Although such research might seem ethically questionable, it is similar to military operations and might be applied to some of these problems (e.g., drone surveillance and drone strikes). Second, the use of such a model in a real‐life application would depend on the amount and quality of information and input data, and thus should not be usable for robbers.

## 6 Dissimilar routing problems

A very restrictive definition of inconsistency might not be desirable, as it allows for a high predictability as soon as parts of the solution are observed. Some of the strictest forms of route‐inconsistency are found in inconsistent routing, and, while not all might be of practical interest, the area in itself provides valuable ideas.

In the literature, inconsistent routing is covered by three methodological concepts: k‐dissimilar VRP (kd‐VPR), m‐peripatetic TSP (m‐PTSP), and m‐peripatetic VRP (m‐PVRP). Both the kd‐VRP and the m‐PVRP aim for a set of routes that include the TSP and VRP constraints: all customers are visited only once; all vehicles start and end at the depot, and the capacity of the vehicle is not exceeded (Talarico et al., 2015b). However, the differences between these concepts are the following: (i) in the m‐PTSP or m‐PVRP, the edges can only be used in one specific period, while in the kd‐VRP, the multiple usage of an edge is limited by similarity constraints; (ii) the m‐PVRP minimizes total cost over all periods, while the kd‐VRP aims for solutions, where the worst‐case cost (over all periods) is minimal (Talarico et al., 2015b). Another interesting aspect of peripatetic models is that, for safe and secure routing problems, the assumption that edges are not allowed to be traversed more than once might be too restrictive.

An alternate modeling approach is routing problems on multi‐graphs, also denoted as road networks. In such an approach, not only the shortest paths but also sub‐optimal paths and more detailed network information are taken into account. An overview of positive effects of this modeling approach and a recent state‐of‐the art survey have been provided by Ben Ticha et al. (2017). Obviously, these multi‐graphs can be useful in the field of inconsistent routing. Related literature on multi‐graphs is discussed in Section 7.

Although m‐PVRP and kd‐VRP problems in the field of CIT, hazardous material, and patrol routing are discussed in Sections 3, 3.1, 3.2, 4, 4.1, 4.2, 5, Table 6 lists the methodological contributions to this problem class. Additionally, we point the reader to studies that we deem to be most relevant for inconsistent routing on multi‐graphs. The second column indicates whether the m‐PTSP, the m‐PVRP, the kd‐VRP, or a multi‐graph is considered. The third and fourth columns indicate the solution method as well as additional characteristics.

**Table 6:** Methodological contributions for the m‐PTSP, the m‐PVRP, the kd‐VRP; references for routing problems on multi‐graphs

Reference	Problem class	Method	Additional
Ageev and Pyatkin (2008)	m‐PTSP	AA	2‐PTSP, metric weights
Ageev et al. (2007)	m‐PTSP	AA	2‐PTSP‐max
Baburin et al. (2009)	m‐PTSP	AA	2‐PTSP, special weights
Kort (1992)	m‐PTSP	H, LB	2‐PTSP
Kort (1993)	m‐PTSP	LB, B&B	2‐PTSP
Kort and Volgenant (1994)	m‐PTSP	/	2‐GTSP
Duchenne et al. (2005)	m‐PTSP	B&C	
Duchenne et al. (2007)	m‐PTSP	B&C	
Duchenne et al. (2012)	m‐PTSP	B&C, B&P	
De Brey and Volgenant (1997)	m‐PTSP	TE	2‐PTSP, diverse special weights
Gimadi et al. (2009)	m‐PTSP	AA	2‐PTSP, special weights
Gimadi and Ivonina (2012)	m‐PTSP	AA	2‐PTSP‐max, special weights
Gimadi et al. (2014)	m‐PTSP	AA	2‐CTSP, 2‐CTSP‐max, special weights
Gimadi et al. (2015)	m‐PTSP	AA	Asymmetric 2‐PTSP & m‐PTSP
Gimadi (2015)	m‐PTSP	AA	m‐PTSP
Gimadi et al. (2016)	m‐PTSP	AO	m‐PTSP
Gimadi and Tsidulko (2017)	m‐PTSP	AA	m‐PTSP
Gimadi and Tsidulko (2019)	m‐PTSP	AO	m‐PTSP‐max, Euclidian
Glebov and Zambalaeva (2012a)	m‐PTSP	AA	2‐PTSP‐max
Glebov and Zambalaeva (2012b)	m‐PTSP	AA	2‐PTSP, special weights
Glebov et al. (2015)	m‐PTSP	AA	Asymmetric 2‐PTSP‐max
Krarup (1975)	m‐PTSP	/	/
Ngueveu et al. (2010a)	m‐PVRP	Hybrid‐TS	
Ngueveu et al. (2013)	m‐PVRP	CG	
Shenmaier (2014)	m‐PTSP	AA	m‐PTSP‐max
Talarico et al. (2015b)	kd‐VRP		
Zajac (2016)	kd‐VRP		
Zajac (2018)	kd‐VRP		
Alinaghian and Naderipour (2016)	multi‐graph		
Behnke and Kirschstein (2017)	multi‐graph		
Fleischmann (1985)	multi‐graph		
Garaix et al. (2010)	multi‐graph		
Huang et al. (2017)	multi‐graph		
Lai et al. (2016)	multi‐graph		
Liu et al. (2014)	multi‐graph		
Letchford et al. (2014)	multi‐graph		
Ben Ticha et al. (2017)	multi‐graph		
Ben Ticha et al. (2019)	multi‐graph		
Setak et al. (2015)	multi‐graph		
Setak et al. (2017b)	multi‐graph		
Setak et al. (2017a)	multi‐graph		
Tikani and Setak (2019)	multi‐graph		
Fröhlich et al. (2020)	multi‐graph		

AA, Approximation Algorithm; AO, Asymptotically Optimal algorithm; B&C, Branch and Cut; B&P, Branch and Price; CG, Column Generation; CVRP‐PD‐TW, Capacitated VRP with Pickup and Delivery and Time Windows; H, Heuristic method; kd‐VRP, k‐dissimilar VRP; LB, Lower Bound; max, Maximize objective; m‐CTPSP, m‐Peripatetic Capacitated Traveling Salesperson Problem; m‐PTSP, m‐Peripatetic Traveling Salesperson Problem; m‐PVPR, m‐Peripatetic Vehicle Routing Problem; TE, Tailor‐made Exact method; TS, Tabu Search.

Table 6 reveals that the m‐PTSP and the m‐PVRP have been intensively studied in the literature. However, most of the research regarding the m‐PTSP focuses on special cases such as the 2‐PTSP, and several of them aim at minimizing costs. Commonly found methods are approximation algorithms, which are algorithms that deliver a solution within a given bound of the best solution with only polynomial run times. More details about the non‐m‐PTSP are described in Section 5, for example, Duchenne et al. (2005, 2007, 2012), where results for using B&C and B&P on the m‐PTSPs have been provided.

## 7 Modeling of multi‐graphs

Many routing problems consider only the shortest (or best) paths between nodes. In the case of arcs with different characteristics (e.g., costs and safety), there might not exist a single best path between two nodes. This is the case for most security problems. Two possibilities to address this fact are the use of either an arc‐based formulation or a multi‐graph. Therefore, an excerpt of relevant publications for the field of security routing is given in the following paragraphs.

**Fig. 9:**
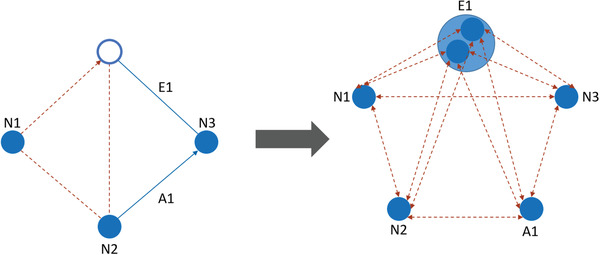
Conversion of a NEARP into a VRP.

**Fig. 10:**
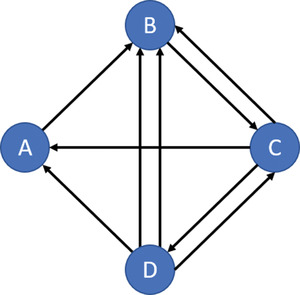
An example of a multi‐graph with two arcs going from vertex D to vertex B.

Problems on road networks are often transformed into VRPs by converting every node, edge, and arc to be serviced into a vertex. The shortest path is calculated between each pair of vertices in both directions, where edges can be traversed in both directions. Thus, a simple‐graph is created. Figure 9 depicts such a transformation of an NEARP into a VRP. The full nodes, edges, and arcs on the left side require service and are all converted to vertices. Due to the orientation flexibility of the edge, it is converted into a vertex with two sub‐vertices. The dashed, double‐sided arrows on the right do not represent edges and, instead, represents two arcs going in opposite directions. However, since most risk definitions are either directly (e.g., arc usage) or indirectly (e.g., arrival times) influenced by the chosen path between vertices, a valid approach for security routing is to model a multi‐graph, which implies that arcs with the same orientation can exist between two vertices. A multi‐graph is depicted in Fig. 10 with two arcs going from vertex D to B.

Garaix et al. (2010) point out that conversion into a simple‐graph VRP can lead to a loss of optimality when multiple attributes such as cost and travel time are defined on the arcs. Hence, they model a dial‐a‐ride problem and solve it with a heuristic and an exact B&P. In both the cases, they solve underlying fixed sequence arc selection problems that determine the best path for the given solution. Furthermore, they compare their results related to the fixed‐sequence arc selection problems with the results on simple graphs for both artificial and real‐world instances. The B&P from Garaix et al. (2010) is further improved by Letchford et al. (2014). The authors develop a pricing for elementary and nonelementary routes for a VRPTW, which takes advantage of the sparse graphs.

Similar results as the ones from Garaix et al. (2010) regarding the benefit of using a multi‐graph can be found in Lai et al. (2016), where artificial instances are used, as well as in Ben Ticha et al. (2017, 2019) that is based on RWIs. In Lai et al. (2016), a time‐constrained heterogeneous VRP is studied. The results from a TS with a FSASP and an MIP are compared, and managerial insights regarding the transportation cost savings due to the multi‐graph are provided. In Ben Ticha et al. (2017), an empirical analysis of multi‐graphs for a real‐world setting is performed. The authors examine a VRPTW, which is solved via B&P on a multi‐graph and two simple graphs for the cheapest paths as well as for the least time‐consuming paths. Several studies show that RWIs with multi‐graphs are tractable. However, Ben Ticha et al. (2017) highlight some limitations and concerns. In particular, computational times increase considerably and correlation between costs and travel times exist, reducing the effect of the achieved benefits. In Ben Ticha et al. (2019), they furthermore develop an ALNS for the problem. In this paper, the approach by Garaix et al. (2010) is improved by a double‐sided labeling approach. The authors compare the results of their heuristic on the multi‐graph to their optimal results provided in Ben Ticha et al. (2017). The ALNS outperforms the B&P on the simple graphs, which emphasizes the benefit of multi‐graphs.

The usage of multi‐graphs can also be found in literature on emission‐based routing. This likely stems from the fact that emission‐efficient paths depend on the load of vehicles and travel times. The latter, however, might change due to congestions during the day. Generally, these studies support the benefits of multi‐graphs. However, since they are not within the scope of our survey, we do not provide details on these studies. Interested readers can refer to Setak et al. (2015, 2017b) where time‐dependent VRPs (TDVRP) are studied. Both of them are modeled on multi‐graphs with given sets of alternative paths between the vertices. Liu et al. (2014) propose a TDVRP with a heterogeneous fleet and apply a GA to generate results. Another TDVRP is studied in Alinaghian and Naderipour (2016) with a focus on modeling fuel consumption. In Huang et al. (2017), an MILP is applied to a deterministic and two‐stage stochastic model of a TDVRP. Behnke and Kirschstein (2017) focus on an emission‐minimizing VRP with heterogeneous vehicles. The authors show how to generate all emission‐minimal paths and apply an MILP to solve the problem.

Soriano et al. (2020) study the effect of using a multi‐graph for a periodic VRP with time spread constraints on services in the context of safe and secure routing. Fröhlich et al. (2020) propose an NEARP in which costs and route consistency are to be minimized. The authors compare their results for artificial as well as RWIs on a simple and a multi‐graph. Improvements can be observed up to a certain instance size. For larger instances, the inherent complexity of the multi‐graph does not allow to benefit from working on the multi‐graph. In Tikani and Setak (2019), a TDVRP in the context of disaster relief operations is studied. The authors aim at minimizing the delays in delivering prioritized items while requiring routes to stay sufficiently reliable. They approach their nonlinear model with three different methods and conclude that using a multi‐graph yields significant improvements.

## 8 Conclusion

The focus of our review is on VRPs with safety and security aspects. The three largest, distinct subgroups were CIT operations, patrol routing, and the transportation of hazardous materials. Usually, connectivity between them is not well highlighted in individual publications, and a review did not exist prior to our work. In our opinion, however, major benefits and further improvements in these fields can be achieved when sharing knowledge gained in the individual studies. As such, we aimed to (i) give an extensive overview of the individual fields and analyze what risk measures, methods, objectives, and constraints are commonly found, (ii) provide suggestions for promising adaptations/expansions for the problems, (iii) enable sharing of knowledge from areas with researchers in this field, and (iv) and give information on the instances used within safe and secure routing.

We did this by, first, giving a brief overview of the most common classifications and definitions that can be found, which may prove to be especially helpful for researchers who might be just starting in this area. Afterwards, details pertaining to hazardous material transportation, patrol routing, and CIT operations are presented. Our focus is mainly on the models and their more distinct characteristics, along with the methods used and the most valuable findings. For each of these areas, after summarizing the literature, we have also proposed possible extensions.

One such extension would be a unification of the used instances. The instances in different publications are very diverse; many have considered a case study. A problem associated with such case studies is the underlying data: (1) crimes prevented due to routes being unpredictable or police presence being high cannot be directly measured; (2) such incidents are rare, making data measurement less reliable; (3) data of security companies is often sensitive and not willingly shared without certain nondisclosure agreements. We suggest to tackle this unification by generating new benchmark instances of complex and realistic problems (e.g., dynamic travel times, time windows, random congestion, and alarms). Solutions for less restricted problems (e.g., ignoring dynamic travel times) could then be evaluated for the master problem with all characteristics. This might provide insight in whether certain characteristics need to be considered for good solutions. Special care should be taken to examine what factors truly affect the likelihood of specific crimes and to what degree. A different approach would be the development of methods aiming to, instead of preventing crimes, perform them. Such methods should be dynamic in order to be able to adapt to the routes of security companies and used as a simulator for testing methods that aim at preventing crimes.

A second consideration for future research paths might be the adaptability of methods and how to handle disruptions or other changes to the system, with one of the most commonly observed one being traffic jams. However, disruptions might also be caused by reactions to sudden incidents and leave formerly well‐covered areas less protected. If something like that could be easily observed, it would allow for undesired exploitation.

Since dissimilar routing problems, while currently not being too present in safe and secure routing, also deliver possibly important insights and methods, we have also included this problem class by presenting a brief summary of the relevant literature. Finally, we also looked at multi‐graph modeling in more details as they can be a compromise between arc‐based formulations, which are high in complexity but allow for more route inconsistencies, and node‐based formulations, with the inverse attributes.

We hope to enable and encourage other researchers to follow these suggestions or gain new ideas and investigate this academically challenging, yet practically relevant field.

## Footnotes

1 Nondominated solutions are those for which no other solution exists that is equally good or better regarding all objectives and better in at least one.

## Appendix

We have provided a brief summary of the used instances in the papers from Tables 3, 4, and 5. Instances that are well known are abbreviated according to the common prefix/suffix. Other instances are described via *RWI* (RWIs), *RGI* (randomly generated instances), and *Art. inst*. (artificial instance). Although all instances could be seen as artificially created, we classified instances that are built strongly on real‐world data (e.g., a specific street network, demands given by companies) as RWIs. Instances that do not belong to the RWI category and that had some random generation mechanism (e.g., random node placement, random distances) are classified as randomly generated instances or RGI. The rest was declared as “Art. inst.” Due to many RWIs being very specific, we have omitted their descriptions and would like to point the interested reader to the specific articles, instead.

### A.1. Instances

- sol, Solomon (1987): For the VRPTW with Cartesian coordinates. Instances have either (i) 25, 50, or 100 customers, (ii) narrow or wide time windows, and (iii) have customers clustered, randomly distributed, or a mix thereof.
  – sol for hazmat, Ma et al. (2012): They were extended with population exposure for arcs according to a grid network, where neighboring grids had positively correlated population sizes.
  – sol for patrol routing, Yassen et al. (2017): Four were simulated with each containing 50 hotspots and five patrols, with two having randomly distributed hotspots and two having clusters.
  – sol for cash‐in‐transit, Michallet et al. (2014): The instances with 100 customers were extended for the periodic VRP with time spread constraints by adding multiple periods and a spread value ε.
  – sol for military patrol routing, Margolis et al. (2019): Two instances based on sol were created for the multi‐vehicle covering tour problem with time windows. A subset of 20 waypoints was selected, and some of the other 80 customers were randomly set as targets.
- kelly, Golden et al. (1998): For the VRP, with Cartesian coordinates. Instances have 200–483 customers. Some instances also have a limitation on tour lengths.
- tai, Taillard (1993): For the VRP, with Cartesian coordinates. Instances have either 75, 100, 150, or 385 customers.
- egl, Li and Eglese (1996): For the CARP. Instances have either (i) 77 or 140 nodes, (ii) one of three combinations of fleet and vehicle capacity, and (iii) sometimes just required arcs. Required arcs are between 51 and 190.
  – md‐egl, Chen et al. (2018): For the min–max multiple depot rural postperson problem. Demands were dropped from egl, and 10 additional depots were evenly added across the instances.
- vrpnc, Christofides et al. (1979): For the VRP with Cartesian coordinates. Instances have either 50, 75, 100, 120, 150, or 199 customers.
- TSPLIB, Reinelt (1991): A set of several different traveling salesman problem with lower and upper bounds.
- A, Augerat et al. (1995): For the VRP with Cartesian coordinates. Instances have between 31 and 79 customers.
- B, Augerat et al. (1995): For the VRP with Cartesian coordinates. Instances have between 30 and 77 customers.
- F, Fisher (1994): For the VRP with Cartesian coordinates. Instance have 44, 71, or 134 customers.
- L, Talarico et al. (2017a): For the risk‐constrained cash‐in‐transit VRP with multiple depots and Cartesian coordinates. They are a modification of the multi‐depot VRP instances by Cordeau et al. (1997) and have between 48 and 360 nodes.
- O, Talarico et al. (2017b): For the risk‐constrained cash‐in‐transit VRP with Cartesian coordinates. Instances have between 9 and 336 customers and were generated in such a way that the optimal solutions were known ahead of time. Tours take the shape of an obelisk.
- P, Augerat et al. (1995): For the VRP with Cartesian coordinates. Instances have between 15 and 100 customers.
- R, Talarico et al. (2015b): For the risk‐constrained cash‐in‐transit VRP with Cartesian coordinates. Instances have (i) between 3 and 19 customers, (ii) one of five risk levels, (iii) demands generated with one of four standard deviations, and (iv) one of five risk thresholds.
- S, Talarico et al. (2017b): For the risk‐constrained cash‐in‐transit VRP with cartesian coordinates. Instances have between 9 and 336 customers and generated in way that the optimal solutions became known ahead of time. Tours take the shape of a spiral.
- V, Talarico et al. (2015b): For the risk‐constrained cash‐in‐transit VRP. They were modified from the VRP library by adding one of five different risk threshold for each. They have between 21 and 300 customers.
- X, Uchoa et al. (2017): For the VRP with Cartesian coordinates. Instances have (i) between 100 and 1000 customers, (ii) have customers clustered, randomly distributed, or a mix thereof, (iii) have the depot placed in the center, in a corner, or randomly, and (iv) one of five demand distributions.
- Bula et al. (2017): Instance from Golden et al. (1984) and Taillard (1999) for the heterogeneous fleet VRP are modified to include risk for the traversed arcs.
- Zografos and Samara (1989): A network consisting of 16 nodes for the tri‐objective location routing problem.
- Capar et al. (2015): A set of 480 randomly generated instances with 20 for each combination of (i) 10, 20, or 40 hotspots and (ii) one to eight state trooper cars.
- Chen (2012): A 4x4‐grid for patrol routing with randomly generated patrol rewards for each of the 80 time units and areas is used.
- Hsieh et al. (2015): A 8x8‐grid for patrol routing with 16 nodes to be patrolled between 1 and 3 times is examined.
- Keskin et al. (2012): A set of 480 randomly generated instances with 20 for each combination of (i) 10, 20, or 40 hotspots and (ii) one to eight state trooper cars.
- Sugiura et al. (2015): A 3x3 grid for patrol routing with 48 links that have to either be patrolled once or twice is examined.
- Duchenne et al. (2005): A set of 60 randomly generated instances with five for each combination of (i) 10, 20, 30, 40, 50, and 60 nodes and (ii) random distances or Euclidean distances.
- Duchenne et al. (2007): A set of randomly generated instances ranging from 10 to 190 nodes, and Euclidean distances is used.
- Duchenne et al. (2012): A set of randomly generated instances ranging from 10 to 120 nodes, and Euclidean distances is used.
